# Successful treatment of acute sickle cell intrahepatic cholestasis with therapeutic plasma exchange

**DOI:** 10.1002/jha2.150

**Published:** 2021-01-06

**Authors:** Dimitris A. Tsitsikas, Rhys Hall, John Meenan, Funmilayo Orebayo, Oloruntoyin Bello‐Sanyaolu, Saket Badle, Manisha Sharma, Susan Jain, Jun Liong Chin

**Affiliations:** ^1^ Department of Haematology Homerton University Hospital NHS Foundation Trust London UK; ^2^ Department of Biochemistry Homerton University Hospital NHS Foundation Trust London UK; ^3^ Intensive Care Unit Homerton University Hospital NHS Foundation Trust London UK; ^4^ Department of Gastroenterology and Liver Medicine Homerton University Hospital NHS Foundation Trust London UK

Acute sickle cell intrahepatic cholestasis (SCIC) is a potentially life‐threatening complication of sickle cell disease (SCD) characterised by extreme hyperbilirubinaemia in the absence of significant obstruction by gallstones, and is caused by extensive sickling within the hepatic sinusoids leading to ischaemia and damage of hepatocytes with ballooning and intracanalicular stenosis and cholestasis. Left untreated, it can cause progressive liver damage and ultimately overt liver failure [1]. Even though there is no robust evidence on the benefit of any therapeutic intervention [2], red cell exchange (RCE) is widely accepted as the mainstay of treatment as it can greatly improve clinical outcomes [3].

We have treated a 30‐year‐old man with sickle cell anaemia (haemoglobin [Hb] SS) and a background of recurrent vaso‐occlusive crises (VOC) as well as a recent episode of life‐threatening post transfusion hyper haemolysis syndrome (PTHS). He had recently agreed to start treatment with hydroxycarbamide. On this occasion, he was admitted with a VOC with generalised bone pains and right upper quadrant pain. His condition deteriorated with intractable pain that could not be controlled using subcutaneous morphine through a patient‐controlled analgesia (PCA) pump at a dose of 4 mg bolus (10‐minute lockout time) with 3 mg background, and he was transferred to the intensive care unit for more aggressive analgesia. Despite being put on an oxycodone intravenous PCA and also receiving intravenous ketamine, his pain remained very difficult to control. His bilirubin was significantly elevated at 635 umol/L with a 50% unconjugated fraction. His alkaline phosphatase, alanine transaminase (ALT), aspartate transaminase (AST), lactic dehydrogenase (LDH) and serum ferritin were also elevated at 427 u/L, 243 u/L, 158 u/l, 467 u/L and 6234 ug/L respectively. His serology was negative for hepatitis A, B, C or E, and an autoimmune screen was unremarkable save for a positive Antinuclear Antibodies with a titre of 1:80. He was afebrile, there was no evidence of any other system dysfunction, and his coagulation profile and platelet count were normal. An abdominal ultrasound showed prominence of the intrahepatic biliary ducts. Subsequently, magnetic resonance cholangiography identified a distal common bile duct stone with some upstream bile duct dilatation. However, the identified stone was not considered sufficient to explain the extreme hyperbilirubinaemia, and a diagnosis of SCIC was made. Given the previous episode of severe PTHS, RCE, that would normally be the treatment of choice, could not be employed unless the condition became imminently life‐threatening.

As the bilirubin was continuing to rise with a peak value of 1126 umol/L, with accompanying evidence of severe systemic inflammation and the ferritin reaching a value of 20,306 ug/L, we decided to try therapeutic plasma exchange (TPE). The aim was primarily to remove bilirubin from the circulation but also circulating pro‐inflammatory cytokines and free Hb in an attempt to dampen the systemic inflammatory response and halt the sickling process.

TPE was performed using the Spectra Optia apheresis system (TerumoBCT) by nurse specialists trained and signed competent to perform the procedure. Five procedures on consecutive days were performed replacing 1.5 plasma volumes for the first 3 and 1 plasma volume for the last 2, using fresh frozen plasma as replacement fluid. Pre‐and post‐samples were obtained to estimate liver function tests, coagulation profile and inflammatory markers such as serum ferritin and LDH. TPE was well tolerated with no observed adverse events.

His pain started improving after the first procedure and continued to improve reducing gradually his analgesia requirements. The bilirubin dropped by 48% after the first procedure, and after the second, there was no significant increment between procedures hence we reduced to 1 plasma volume for the last 2. By the fifth procedure, it had stabilised to values less than 100 umol/L, corresponding to the patient's steady state. Ferritin dropped by 65% after the first TPE and continued to drop subsequently with no increments between procedures (Figure 1). ALT and AST normalised after the first and second procedures respectively and remained within normal levels thereafter. His clotting screen, platelet count and renal function remained normal throughout. His Hb that had dropped from 102 g/L on admission to 82 g/L before commencing TPE dropped further to 59 g/L after the third procedure at which point he was commenced on erythropoietin and iron supplements. After cessation of TPE his bilirubin and inflammatory markers remained stable while a repeat ultrasound of the liver performed 2 weeks after the first one showed resolution of the previously observed intrahepatic biliary duct dilatation. He was discharged 6 days after completion of TPE. On review 1 and 2 weeks post‐discharge, he was well with a bilirubin of 50 and 55 umol/L, respectively.

**FIGURE 1:**
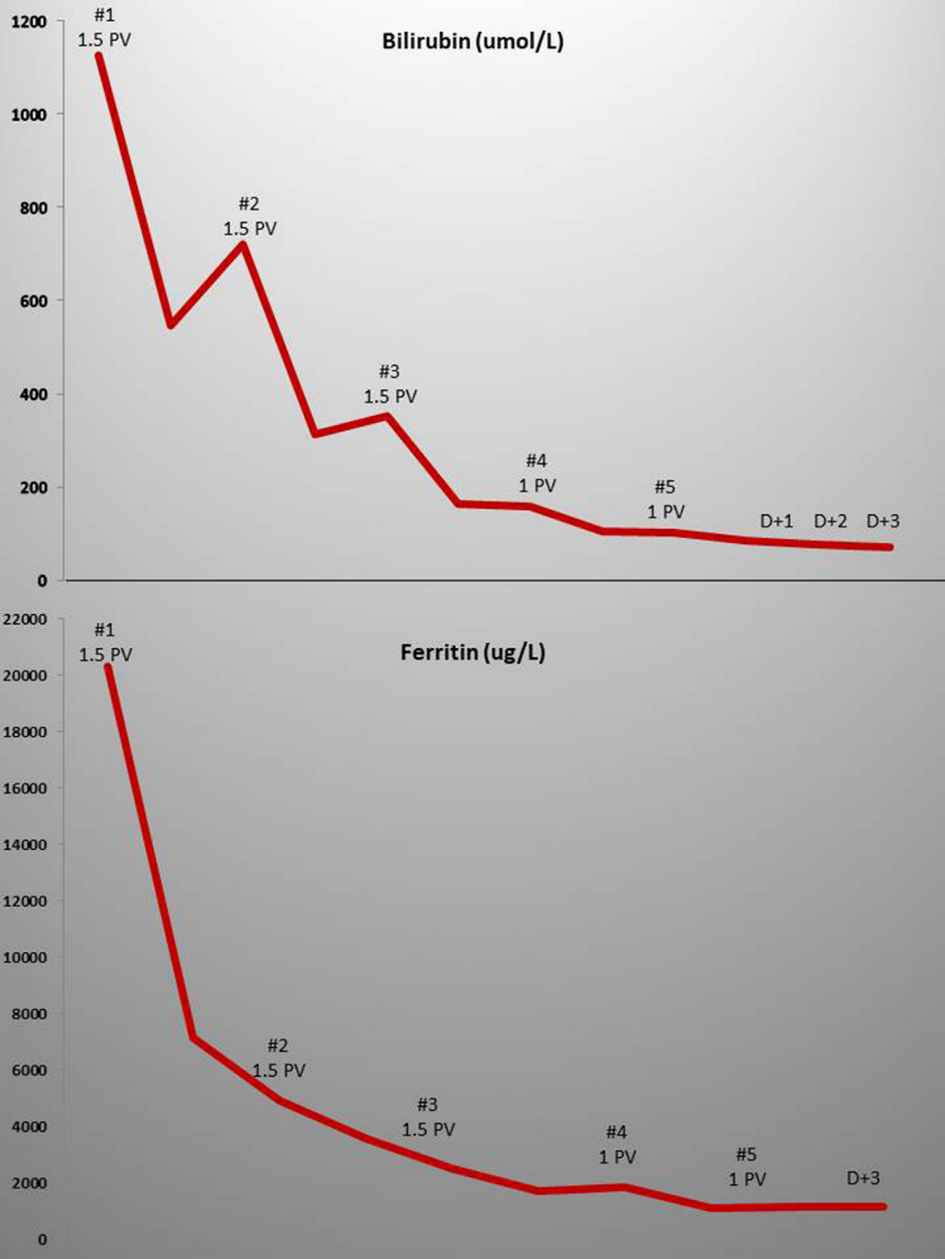
Biochemical response to therapeutic plasma exchange

SCD is associated with a systemic inflammatory state at least partly due to ongoing limited bone marrow infarction and release of fat in the venous circulation with generation of pro‐inflammatory cytokines under the action of secretory phospholipase A_2_ even at steady state, a situation accentuated during crises [4]. Furthermore, such cytokines are considered to play an important role in the pathogenesis of the acute chest syndrome and cause tissue damage leading to chronic sickle lung disease [5, 6]. TPE has the potential to remove a whole host of harmful substances such as free Hb, inflammatory cytokines and prothrombotic proteins from the circulation [7], and it has been used successfully as an adjunct to RCE for treatment of multiorgan failure [8] and ACS [9] while we have advocated its use in the acute management of fat embolism syndrome in SCD after adequate RCE [10].

This is the first reported case of TPE used as monotherapy without the use of red cell transfusion for acute SCIC or indeed any other acute sickle‐related complication, and the dramatic biochemical, radiological and sustained clinical improvement we observed shows that TPE can lead to rapid improvement of the systemic inflammation during a sickle crisis and that its role as an adjunct to RCE or as an alternative when the latter is contraindicated needs to be further explored as a potentially important intervention to be added to our armamentarium in managing complications of SCD.

## CONFLICT OF INTEREST

The authors declare that there is no conflict of interest that could be perceived as prejudicing the impartiality of the research reported.

## AUTHOR CONTRIBUTIONS

Dimitris A. Tsitsikas designed the project and wrote the paper; Rhys Hall and John Meenan coauthored the paper; Funmilayo Orebayo, Oloruntoyin Bello‐Sanyaolu and Saket Badle collected data and critically reviewed the manuscript; and Manisha Sharma, Susan Jain and Jun Liong Chin co‐designed the project and critically reviewed the manuscript.
